# Antisense Therapy in Neurology

**DOI:** 10.3390/jpm3030144

**Published:** 2013-08-02

**Authors:** Joshua J.A. Lee, Toshifumi Yokota

**Affiliations:** 1 Department of Medical Genetics, Faculty of Medicine and Dentistry, University of Alberta, 8812-112 St, Edmonton T6G 2H7, Canada; E-Mail: jjlee@ualberta.ca; 2 The Friends of Garrett Cumming Research and Muscular Dystrophy Canada HM Toupin Neurological Science Research Chair, 8812-112 St, Edmonton T6G 2H7, Canada

**Keywords:** Duchenne muscular dystrophy (DMD), Fukuyama congenital muscular dystrophy (FCMD), myotonic dystrophy (DM), spinal muscular atrophy (SMA), Huntington’s disease (HD), amyotrophic lateral sclerosis (ALS), limb-girdle muscular dystrophy 2B (LGMD2B), Miyoshi myopathy (MM), distal myopathy with anterior tibial onset (DMAT), antisense therapy

## Abstract

Antisense therapy is an approach to fighting diseases using short DNA-like molecules called antisense oligonucleotides. Recently, antisense therapy has emerged as an exciting and promising strategy for the treatment of various neurodegenerative and neuromuscular disorders. Previous and ongoing pre-clinical and clinical trials have provided encouraging early results. Spinal muscular atrophy (SMA), Huntington’s disease (HD), amyotrophic lateral sclerosis (ALS), Duchenne muscular dystrophy (DMD), Fukuyama congenital muscular dystrophy (FCMD), dysferlinopathy (including limb-girdle muscular dystrophy 2B; LGMD2B, Miyoshi myopathy; MM, and distal myopathy with anterior tibial onset; DMAT), and myotonic dystrophy (DM) are all reported to be promising targets for antisense therapy. This paper focuses on the current progress of antisense therapies in neurology.

## 1. Introduction

Antisense oligonucleotides (AOs) are short, synthetic nucleic acid sequences that selectively hybridize to target sequences in messenger RNA (mRNA). AOs can cause inhibition or redirection of splicing and inhibition of protein synthesis through various mechanisms, including disruption of the cell’s splicing machinery, interference with the ribosomal complex, and/or by activation of RNase H1-mediated degradation of the oligo-RNA heteroduplex [1]. Antisense therapy is an approach to fighting diseases using DNA-like molecules (AOs). After initially observing antisense-mediated RNA regulation in nature, investigations using model systems to test the feasibility of using synthetic AOs to reduce levels of specific mRNA transcripts quickly followed. Early experiments showed that AOs were effective in reducing target transcripts and protein synthesis [2]. However, despite promising early results, the use of AOs in disease therapy has been stymied by technical challenges and progress has been slow. Despite more than 20 years of research and clinical investigations, the United States Food and Drug Administration (FDA) has only ever approved two marketable AO drugs, Vitravene (Isis Pharmaceuticals, Carlsbad, CA, USA), for the treatment of cytomegalovirus retinitis in immunocompromized Acquired Immune Deficiency Syndrome (AIDS) patients with human immunodeficiency virus (HIV) infection [3], and, recently, Kynamro® (Isis Pharmaceuticals, Carlsbad, CA, USA) for the treatment of familial hypercholesterolemia. Although approved in 1998, Vitravene was removed from the market in 2004. Notwithstanding its slow progress, antisense remains a widely popular area of research in molecular biology, and with recent advancements in oligo chemistries and promising results from recent clinical trials it may well be that the day of AOs in the clinical arena in neurology is close at hand.

## 2. Challenges

Although promising, the headway of antisense therapy in the clinical realm has been quite slow. To better appreciate the current status of AO drug therapies, it is important to consider the hurdles that AOs have had to overcome. The first of these hurdles is drug delivery. First generation AOs do not easily cross the lipid bilayer of the cell, making intracellular potency via systemic delivery problematic since these AOs cannot readily penetrate to their intracellular targets at significant concentrations to be effective [4,5,6,7]. In the case of certain neurodegenerative diseases, such as Huntington’s disease and Alzheimer’s, the limited permeability of the blood-brain barrier further compounds the difficulty of effective drug administration to target cells of the central nervous system (CNS) [8]. Another problem associated with first generation AOs is off-target toxic effects [9]. DNA and RNA can be immunostimulatory, binding to and activating toll-like receptors or other receptors involved in innate immunity in a sequence- and chemistry-dependent manner [10]. Other biological barriers include uptake and sequestration of AOs in the reticuloendothelial system and intracellular sequestration in oligo-protein complexes and phagolysosomes [11]. Furthermore, to achieve biochemical efficacy, a large proportion of RNA targets must be hybridized and silenced—this number can vary widely, but can be as high as >90 percent [12]. To overcome these challenges, AOs have been designed such that the ribose backbone (normally present in RNA and DNA) is replaced with other chemistries. These constructs are so distinct from classical nucleic acid structures that they are not readily targeted by nucleases or DNA/RNA-binding proteins. These modifications result in increased stability and help prevent most off-target toxic effects. The various chemistries and modifications of AOs will be discussed in-depth in the next section. Regarding issues of delivery to CNS tissues, studies have shown the feasibility of AO-mediated RNA silencing in CNS tissues by AO drug administration into cerebrospinal fluid (CSF) via cerebral ventricles and intrathecal injection [13,14]. Drug administration into CSF via cerebral ventricles is a common medical practice in humans [15]. Studies involving administration of AOs into cerebral ventricles have shown significant oligonucleotide concentrations present not only in the brain and brainstem but also in many levels of the spinal cord after delivery in rats and nonhuman primates, providing evidence of delivery efficacy and sidestepping the hurdle of permeating the blood-brain barrier [16].

## 3. Comparative AO Chemistries

**Figure 1:**
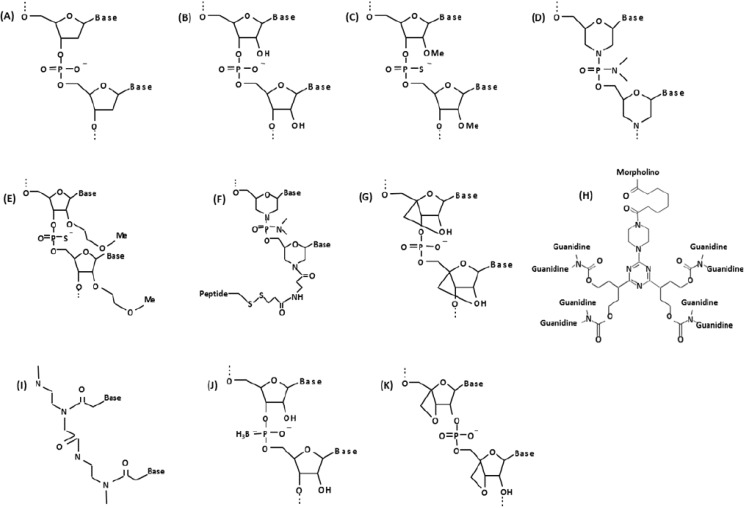
Chemical structure of biological and synthetic oligonucleotides. (**A**) DNA; (**B**) RNA; (**C**) 2'O-methylphosphorothioate (2'O-MePS); (**D**) Morpholino (PMO); (**E**) 2'-methoxyethoxy (2'-MOE); (**F**) PMO with peptide conjugate (PPMO); (**G**) Locked nucleic acid (LNA); (**H**) Vivo-morpholino (vPMO); (**I**) Peptide nucleic acid (PNA); (**J**) Boranophosphate-oligodeoxy-nucleoside (BH3-ODN); (**K**) Oxetane-modified AO.

To avoid nuclease degradation, facilitate stronger base-pairing with target mRNA sequences, increase stability, and enable easier delivery into the cell, a variety of AO chemistries have been developed (Figure 1). One of the most widely used oligo chemistries is the 2'O-methylphosphorothioate- modified (2'OMePS) antisense oligo. These oligos contain a 2'-modification of the ribose ring as well as phosphorothioate linkages throughout their length (Figure 1C). The 2'OMePS AOs exhibit improved stability and increased cellular uptake via conventional delivery reagents. These AOs have also been shown to be very efficient *in vivo* [17,18]. The safety of this particular AO chemistry has been well characterized through a number of preclinical and clinical trials for several diseases [19,20,21]. Of note, the Prosensa/GlaxoSmithKline Duchenne muscular dystrophy (DMD) drug development program (Prosensa Therapeutics, Leiden, the Netherlands, and GlaxoSmithKline, London, UK), currently one of the leading bodies in antisense therapy research, employs this particular antisense chemistry [20,21].

Another oligo chemistry that is gaining in popularity is the phosphorodiamidate morpholino oligomer (PMO, morpholino). The PMO chemistry differs from traditional DNA/RNA chemistry in that the nucleic acid bases are bound to morpholine moieties as opposed to deoxyribose/ribose rings and the phosphodiester backbone is replaced by a phosphorodiamidate linkage [22] (Figure 1D). Like other oligos, the chemical modifications to PMOs render them sufficiently different from conventional nucleic acid chemistries so that they are not recognized by nucleases, making them very stable. Advantages of PMOs include increased binding efficiency to RNA targets and insusceptibility to metabolic degradation. Moreover, PMOs do not activate toll-like receptors, the nuclear factor (NF)-κB-mediated inflammatory response, or the interferon system [23]. Currently a phase 2 clinical trial involving PMOs for Duchenne muscular dystrophy is being conducted by Sarepta Therapeutics (Cambridge, MA, USA), and a significant improvement in 6-min walking distance (6-min walk test) has already been reported (MDA conference presentation, Washington, April 2013).

There are several groups of next generation antisense compounds that have shown very promising results in animal models. For example, 2'-methoxyethoxy (2'-MOE)-modified oligonucleotides containing lipophilic 2'-O-alkyl-substituted nucleobase modifications demonstrate high RNA binding affinity and metabolic stability, and can be used as gapmers to catalyze RNase H1-mediated degradation of target nucleic acids [24,25,26] (Figure 1E). 2'-MOE oligos have been used *in vivo* to target toxic mRNA triplet repeats in myotonic dystrophy [27]. Vivo-morpholinos (vPMOs) are octa guanidine (cell-penetrating moiety) conjugated PMOs (Figure 1H) and have shown very efficient splicing modulation in studies targeting the *FCMD* gene, *DMD* exons 6 and 8 multi skipping in dystrophic dogs, and exons 45–55 in *mdx52* mice [28,29,30]. PMOs with peptide conjugates (PPMOs or PMOs with muscle targeting peptides; Figure 1F) act similarly to vPMOs and efficiently rescued cardiac muscle as well as skeletal muscles in *mdx* mice [31,32,33,34,35,36,37]. Peptide nucleic acids (PNAs) are another class of antisense oligo in which the phosphodiester-linked deoxyribose/ribose backbone is replaced by peptide-linked repeating N-(2-aminoethyl)-glycine units, to which the nucleobases are attached [38] (Figure 1I). PNAs exhibit greater binding strength than many other AOs and are extremely stable, though their solubility in water is much lower [39,40]. Locked nucleic acid (LNA) AOs contain a 2'-C, 4'-C-oxymethylene-linkage which “locks” the deoxyribo/ribo sugar structure in an N-type conformation [41] (Figure 1G). LNAs are stable against exonucleolytic degradation, exhibit high thermostability and hybridize strongly with target nucleic acids [42,43]. Several LNA analogs have been developed [42,44]. The characteristics of LNA constructs have made them the oligo of choice for several molecular applications, including microarrays [45], genotyping assays [46,47,48], and for the stabilization of DNA triplex formation in gene silencing [49]. In 1992, Sood *et al*. first reported an antisense oligo chemistry containing a boronated phosphate backbone (boranophosphate) [50]. Known as boranophosphate-oligodeoxy-nucleosides (BH3^−^-ODN), these AOs differ from classical DNA/RNA constructs in that they contain a borane group in place of a non-bridging oxygen species in the phosphodiester backbone (Figure 1J). Boranophosphates have been shown to activate RNase H1-mediated RNA cleavage [51]. Furthermore, experiments have demonstrated the highly lipophilic nature of boranophosphates [52], thus facilitating their transport across the bilipid membrane to target nucleic acids. This characteristic is likely due to the increased hydrophobicity of BH_3_ compared with oxygen. Boron-modified dNTPs have also been successfully employed in DNA sequencing assays—by taking advantage of the nuclease-resistant nature of boranophosphates [53,54], researchers are able to sequence resultant nucleic acid fragments following exonuclease digestion [55]. Oxetane-modified oligonucleotides (Figure 1K) are another form of AO which have proven their feasibly as antisense molecules by exhibiting resistance to nuclease digestion, the ability to activate RNase H1-mediated cleavage of the AO/RNA heteroduplex, tightly bind to their target nucleic acid sequences, and efficiently silence gene expression *in vitro* [56,57]. Development of more effective and less toxic AOs will be a key to the success of AO therapy.

## 4. Antisense Oligo Delivery

The method of delivery of antisense oligonucleotides in neurology is mainly predicated on the nature of the disease. There are two major targets of delivery: tissues of the central nervous system (CNS) and all other non-CNS tissues. In the case of neurodegenerative diseases such as Huntington’s disease (HD), amyotrophic lateral sclerosis (ALS), and spinal muscular atrophy (SMA), direct targeting of CNS tissues is often desirable and can be accomplished via intrathecal injection, intracerebroventricular administration, and intraparenchymal delivery to the striatum [58,59,60,61]. This sidesteps the hurdle of the blood-brain barrier and increases the likelihood of oligo uptake to desired CNS tissues. A recently concluded phase I clinical trial involving Isis Pharmaceuticals’ antisense drug ISIS 333611 against SOD1 for the treatment of ALS reported no serious adverse effects following intrathecal injection [60].

For the antisense treatment of myopathic diseases, such as Duchenne muscular dystrophy (DMD), systemic administration via subcutaneous or intravenous injection, as well as direct intramuscular injection has been shown to facilitate widespread oligo distribution and effective intracellular uptake [62,63]. In the case of DMD, the preexisting pathology of the muscle tissues further enhances oligo uptake, as the plasma membranes of these muscle cells are unstable and contain small perforations, allowing AOs to more readily penetrate to their intracellular targets [64].

As previously mentioned, the intracellular delivery of AOs is further aided by chemical modifications which allow the oligos to more easily penetrate cell membranes. These modifications come in various forms, such as arginine-rich peptide conjugated morpholinos (PPMOs) or morpholinos linked to octa guanidine dendrimers (vPMOs), but each chemical adduct is designed to aid intracellular uptake.

In some instances, a dualistic targeting of both CNS and non CNS tissues is favorable, especially in cases involving multiorgan diseases. For example, Hua *et al.* demonstrated that liver plays an important role in SMA pathogenesis and were able to show a significant increase in survival in severely affected SMA mice following subcutaneous delivery of AOs. Increased survival following systemic AO administration was more pronounced than intracerebroventricular administration to CNS tissues alone and was further increased when both routes of oligo administration were coupled together [59].

## 5. Antisense Therapy in Neurology: Overview

In the second half of this article, the use of antisense oligos for Duchenne muscular dystrophy (DMD), Fukuyama congenital muscular dystrophy (FCMD), myotonic dystrophy (DM), spinal muscular atrophy (SMA), dysferlinopathy, Amyotrophic lateral sclerosis (ALS), and Huntington’s disease (HD) will be covered. Although they are all targeted by antisense therapy, therapeutic strategies for these disorders are quite different. For example, to target DMD, antisense-mediated exon skipping can remove nonsense mutations or frame-shifting mutations from mRNA [65,66,67]. To treat the mutation in the *FCMD* gene, a cocktail of vivo-morpholino AOs targeting splice enhancer sites and splice silencer sites led to correction of the aberrant splicing pattern in cell and mouse models [29]. RNase H1-mediated degradation of toxic RNA with 2'-MOE antisense for myotonic dystrophy type 1 showed very promising results in the mouse model [68]. A unique “knock up” approach (exon inclusion) targeting the *SMN2* gene with 2'-MOE antisense or PMOs has been used to treat SMA cell and mouse models [69,70]. In the following sections, recent progress of antisense therapy in neurology and remaining challenges will be discussed. 

## 6. Exon Skipping Therapy for DMD

**Figure 2:**
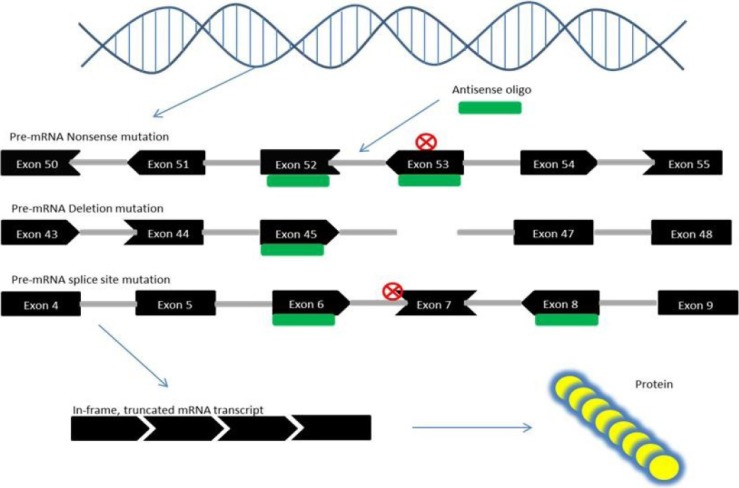
Mechanism of exon skipping therapy for Duchenne muscular dystrophy (DMD). Nonsense mutations in the DMD gene can create a novel STOP codon which results in the loss of DMD protein. Exon skipping corrects this error when exons (black) that are bound to antisense oligos (green) are spliced out of the pre-mRNA, and the resulting exon sequences “fit together”, *i.e.*, are in-frame (denoted by the shape of each exon—ends that fit together are in-frame). Out-of-frame mutations caused by the loss of exonic sequences, through deletion or splice site mutations, can also be corrected through exon skipping, which removes exons adjacent to the mutation site so that the remaining exons are in-frame. The result is a truncated yet partly functional protein, as in the case of Becker muscular dystrophy (BMD).

DMD is an X-linked recessive form of muscular dystrophy, affecting around one in 3,500 boys worldwide, which leads to muscle degeneration and eventual death [71,72]. DMD is caused by mutations in the gene encoding dystrophin [73]. Recently, exon skipping has been heavily researched for the treatment of DMD [74,75]. Exon skipping employs antisense oligos as “DNA Band-Aids” to skip over the parts of the mutated gene that block the effective creation of proteins and restore the reading frame (Figure 2) [76]. In fact, such exon skipping of disease-causing mutations occurs spontaneously in DMD patients and animal models to some extent [77,78,79,80,81]. The efficacy of exon skipping was tested in several animal models including dystrophic *mdx* mice and dystrophic dogs as well as human DMD cells [30,35,82,83,84,85,86,87,88,89,90,91,92,93,94,95]. Systemic rescue of animal models with exon skipping has been demonstrated in dystrophic dogs (exons 6 and 8 multi-skipping), *mdx* mice (exon 23), and *mdx52* mice (exon 51 and exons 45–55 multi-skipping) [17,28,82,96]. Currently, systemic clinical trials are being conducted targeting exon 51 in the *DMD* gene with PMOs and 2'OMePS antisense oligos, and very promising data have already been presented [20,97,98,99,100] (Table 1). Possibly, these antisense drugs will be approved by the Federal Drug Administration (FDA) in the near future. In addition, the first clinical trial of DMD targeting exon 53 skipping will start in Japan scheduled in 2013 (Nippon Shinyaku Co. Ltd. and National Center of Neurology and Psychiatry news release; UMIN-CTR Clinical Trial number UMIN000010964) (Table 1). Remaining challenges include: (1) limited efficacy of AOs, especially in the heart; (2) unknown long-term safety; (3) limited applicability (only approximately 10% of DMD patients can be treated with exon 51 and exon 53 skipping therapy, respectively).

**Table 1:** Current clinical trial status of antisense drugs for use in neurodegenerative and neuromuscular disorders.

Disease	Drug	Chemistry	Mechanism of action	Target	Clinical Phase	Status	Sponsor	Clinicaltrials.gov ID
DMD	Eteplirsen (AVI-4658)	PMO	Exon Skipping	Exon 51	Phase II	Completed	Sarepta Therapeutics	NCT01396239
DMD	Drisapersen (PRO051/GSK2402968)	2'OMePS	Exon Skipping	Exon 51	Phase III	Recruiting	Prosensa Therapeutics/ GlaxoSmithKline	NCT01803412
DMD	NS-065/NCNP-01	PMO	Exon Skipping	Exon 53	Phase I	Recruiting	Nippon Shinyaku Pharmaceuticals	NA
SMA	ISIS-SMNRx	2'-MOE	Exon Inclusion	Exon 7	Phase II	Recruiting	Isis Pharmaceuticals	NCT01839656
ALS	ISIS-SOD1Rx/ISIS 333611	2'-MOE	Gapmer	Exon 1	Phase I	Completed	Isis Pharmaceuticals	NCT01041222
DM1	PRO135	NA	NA	CUG expansion	Preclinical	In progress	Prosensa Therapeutics	NA
HD	PRO289	NA	NA	CAG expansion	Preclinical	In progress	Prosensa Therapeutics	NA

## 7. Splicing Correction Therapy for FCMD

**Figure 3:**
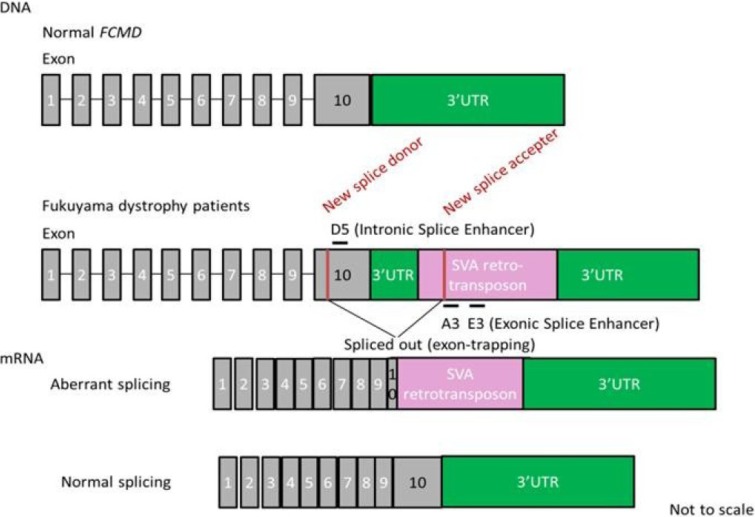
Strategy of antisense therapy for Fukuyama dystrophy. Retrotransposon insertion in the *FCMD* gene leads to aberrant splicing. An antisense vivo-morpholino cocktail (A3, E3 and D5) restores normal splicing.

FCMD is an autosomal recessive form of muscular dystrophy mainly described in Japan [101]. The gene responsible for FCMD encodes a novel protein, fukutin [102]. Fukutin is believed to add chains of sugar molecules (glycosylation) to α-dystroglycan, a member of the dystrophin glycoprotein complex [103,104]. Interestingly, most patients (87%) with mutated *FCMD* gene bear chromosomes that have a 3-kb retrotransposon insertion into the 3'-untranslated region (UTR) of the gene derived from a single ancestral founder [105,106]. The aberrant mRNA splicing induced by the SINE-VNTR-Alu (SVA) retrotransposon exon-trapping is responsible for the pathogenesis of FCMD [29] (Figure 3). The insertion induces splicing errors and cryptic splice site activation with a new splice donor in exon 10 and a new splice accepter in the SVA insertion site. This results in aberrant splicing and truncation of exon 10. To rescue the mutated gene, a cocktail of at least three antisense oligos was required [91]. These oligos were targeted against intronic or exonic splicing enhancer sites (called ISE or ESE). These splicing enhancers are sites with consensus sequences that bind to splicing activator proteins [107,108]. They increase the probability that a nearby site will be used as a splice junction [109]. A cocktail of vPMOs led to normal fukutin mRNA expression and protein production in human patient cells as well as the mouse model *in vivo* [29].

## 8. Antisense Therapy for DM1

**Figure 4:**
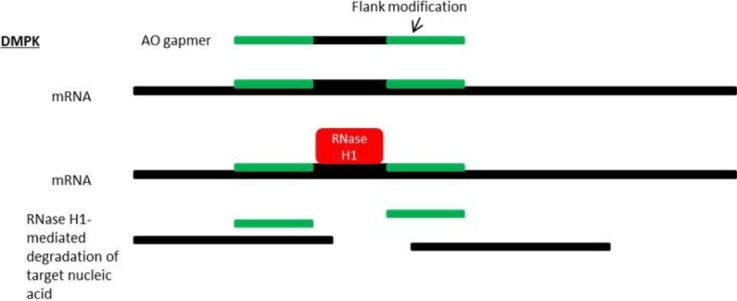
Mechanism of antisense silencing via RNase H1 activity. Myotonic dystrophy (DM1) is caused by RNA gain-of-function due to an expanded CUG repeat in the *dystrophia myotonica-protein kinase* (*DMPK*) gene transcript.RNase H1-mediated degradation of target nucleic acids is facilitated by AO “gapmers”, composed of a central gap region which supports RNase H1 activity and flanking nucleotides at the 5' and 3'-ends which are resistant to RNase H1 degradation and display strong binding affinity for target RNA.

Myotonic dystrophy is the most common adult form of muscular dystrophy and is characterized by myotonia (slow relaxation of the muscles), progressive muscle weakness, and atrophy [110]. DM can also cause dysfunction of heart, eye, and brain tissues, as well as the gastrointestinal and endocrine systems [111,112]. Myotonic dystrophy type 1 (DM1) and myotonic dystrophy type 2 (DM2) are multisystemic microsatellite expansion disorders caused by an expanded CTG tract in the 3' UTR of the *dystrophia myotonica-protein kinase* gene (*DMPK*) and an expanded CCTG tract in the first intron of the *CCHC-type zinc finger, nucleic acid binding protein* gene (*CNBP*, also known as *ZNF9*), respectively [113,114,115,116,117,118]. Disease phenotype (including age of onset and severity) is highly correlated with repeat number. In the case of DM1, unaffected individuals tend to have CTG repeats between 5 and 35 while DM1 patients often present with expansions between 50 and >2,000 [119,120]. DM follows an autosomal dominant pattern of inheritance and, although the precise molecular mechanisms are unknown, symptoms are thought to arise owing to the toxic gain-of-function of RNA transcripts containing expanded repeats, which causes the transcripts to be retained and accumulate in the nucleus [121]. Wang *et al*. have also provided evidence to suggest a possible dominant-negative effect of expansion-containing mutant RNA transcripts [122]. Protein-level gain-of-function is not likely, as the CTG expansion region lies outside of the *DMPK* coding region in the 3' UTR. Antisense-mediated suppression of *DMPK* RNA transcripts is, therefore, a promising therapeutic approach [123,124] (Figure 4). Importantly, there is considerable evidence implicating diminished DMPK transcripts in DM1 pathology, with a consensus among several studies that production and processing of DMPK mRNA is inhibited by expansion-containing mutant transcripts [125,126,127,128,129,130]. In their study utilizing homozygous DMPK-null mice, Reddy *et al*. showed that these mutants develop a progressive myopathy that is pathologically similar to DM, underscoring the importance of DMPK in maintaining proper skeletal muscle condition [131].

Recent *in vitro* studies have helped shed light on the therapeutic efficacy of several AO chemistries targeted against the microsatellite expansion of DM1 [132]. *In vivo* studies using 2'-MOE, LNA, and PPMO chemistries have provided evidence of efficient, long-lasting antisense-mediated knockdown of mutant RNA transcripts, as well as amelioration of physiological and transcriptomic abnormalities in DM1 mouse models [27,133,134]. Researchers from the University of Rochester and Isis Pharmaceuticals, Inc. have developed efficient methods to treat DM1 in a mouse model with systemically administered 2'-MOE modified antisense oligos [27]. They have successfully reversed symptoms of DM1 in these mice by eliminating toxic RNA in muscle fibers. Currently the group is working to improve their lead compound further, developing antisense oligos with stronger efficacy against the toxic RNA, but with minimal toxic effects. 

Currently, no clinical trials are underway which involve AOs for the treatment of DM. Prosensa Therapeutics (Leiden, Netherlands), is currently in the pre-clinical stage of developing an antisense oligo, PRO135, which was shown to ameliorate toxic effects *in vivo* in DM1 preclinical models (Table 1).

## 9. Exon Inclusion Therapy for SMA

**Figure 5:**
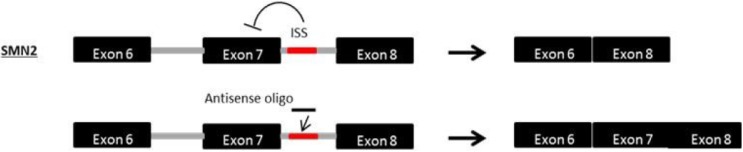
Mechanism of antisense exon 7 inclusion in *SMN2*. Spinal muscular atrophy (SMA) is caused by a loss-of-function mutation in the *SMN1* gene. Within the *SMN2* gene, a paralogue of *SMN1*, a single nucleotide substitution in exon 7 interferes with an exonic splicing enhancer, impairing production of normal SMN protein. AOs targeted to the intronic splice silencer site (ISS) in intron 7 of *SMN2* facilitate the retention of exon 7 within the mature mRNA, increasing the production of functional SMN protein.

Spinal muscular atrophy (SMA) is a lethal autosomal recessive disease caused by a genetic defect in the *SMN1* (*survival motor neuron*) gene [135,136]. SMA is characterized by the deterioration of spinal motor neurons, followed by weakness and wasting of the voluntary muscles in the arms and legs of infants and children, resulting in death during childhood [137]. Interestingly, SMA patients retain at least one copy of a highly homologous gene called *SMN2* [138]. *SMN2*, an inverted duplicate copy nearly identical to *SMN1*, is unable to compensate for the loss of *SMN1* due to a C-T transition in exon 7 which interferes with a splice modulator, causing exon 7 to be lost and rendering the resultant SMN protein nonfunctional; however, some full-length SMN transcripts (~10%) and functional SMN proteins are still produced. The *SMN2* gene differs from *SMN1* by only five base pair changes [139]. Consequently, upregulation of SMN by modification of *SMN2* exon 7 splicing is a promising therapeutic approach (Figure 5), an approach that has already demonstrated favourable results in animal models [69,140,141,142,143]. Antisense PMOs targeting splice silencing motifs that promote exon 7 retention successfully rescued the phenotype in a severe mouse model of SMA after intracerebroventricular delivery [144]. In addition, the PMO injection led to longer survival after a single dosing by ICV injection. 

Recently, Isis Pharmaceuticals, Inc. announced the commencement of an open-label, multiple-dose, dose-escalation Phase II clinical trial which utilizes their antisense oligo drug ISIS-SMNRx (Table 1). The study involves patients with infantile-onset SMA and is currently seeking to recruit eight participants between three weeks and seven months of age in the US and Canada. The aim of the study is to provide information regarding the safety and tolerability of ISIS-SMNRx. The results of this investigation will help lay the foundation for a future large-scale phase II/III clinical trial. The drug under investigation, ISIS-SMNRx, is a 2'-MOE modified AO designed to modulate *SMN2* splicing, thereby increasing levels of SMN protein. A previously concluded Phase I trial evaluating ISIS-SMNRx (ClinicalTrials.gov identifier: NCT01494701) showed the drug to be well-tolerated across all doses and also reported a significant improvement in muscle function in several participants.

## 10. Exon Skipping Therapy for Dysferlinopathy

The dysferlinopathies are a category of muscular dystrophy arising due to mutations in the *dysferlin* (*DYSF*) gene [145,146]. Three clinically distinct autosomal recessive muscular dystrophies are attributed to *DYSF* mutations: limb-girdle muscular dystrophy type 2B (LGMD2B), Miyoshi myopathy (MM), and distal myopathy with anterior tibial onset (DMAT) [147,148,149,150,151,152]. Dysferlinopathy is characterized by progressive muscle weakness and atrophy with onset usually beginning in adulthood and commencing in either the proximal or distal muscles, defining the clinical phenotype. Although distinct initially, the clinical phenotypes of dysferlinopathy include a wide spectrum of pathology that becomes less divergent as the disease progresses, eventually including both proximal and distal muscle groups, becoming one indistinguishable disorder. The sarcolemmal protein dysferlin is a transmembrane protein that is ubiquitously expressed and is found abundantly in cardiac and skeletal muscle where it plays a pivotal role in plasma membrane re-sealing [147,153,154,155,156,157,158,159].

A promising therapeutic approach to treating dysferlinopathies is exon skipping, wherein AOs are used to selectively target exonic sequences and prevent their incorporation into the final mRNA transcript [65,160]. This process of splicing modulation restores the open reading frame and leads to the production of a truncated-yet functional protein and has already been demonstrated *in vitro* using dysferlinopathy patient-derived cells [161]. In addition, Sinnreich *et al*. reported a case wherein a mildly affected mother with two severely affected daughters, both having LGMD2B with homozygous *DYSF* null mutations, was found to carry a lariat branch point mutation that resulted in the in-frame exon skipping of exon 32. The action of the resulting dysferlin protein is thought to account for her mild phenotype [162]. Therefore, at least dysferlin exon 32 is thought to be a promising target of exon skipping therapy, although there are currently no ongoing or pending clinical trials involving AO-mediated therapy for dysferlinopathy.

## 11. Antisense Therapy for Amyotrophic Lateral Sclerosis (ALS)

Amyotrophic lateral sclerosis (ALS) is a progressive neurodegenerative disease affecting upper and lower motor neurons in the brain and spinal cord [163]. Though associated with some clinical heterogeneity, ALS typically manifests during adulthood and is characterized by progressive neuronal death, spasticity, muscle atrophy, paralysis, and death within ~5 years of diagnosis [164,165,166]. Most cases of ALS are sporadic; however, ~10% of cases are familial and follow an autosomal-dominant pattern of inheritance [167,168]—of these, 20% are caused by mutations in the *Cu/Zn superoxide dismutase* (*SOD1*) gene, resulting in a toxic gain-of-function via a currently unknown mechanism [169,170,171]. Although currently believed to be the result of a gain-of-function mechanism, initial investigations into the role of *SOD1* in ALS supported a loss-of-function mechanism [172,173]. Belief in a loss-of-function model waned significantly following *in vivo* experiments involving transgenic mice expressing human SOD1 protein, which exhibited progressive neurodegeneration, mirroring human ALS clinical pathology [170,174,175]. Clinical observations which failed to support a connection between SOD1 activity and disease progression further eclipsed the idea of loss-of-function [176]. However, in their recent article, Saccon *et al*. compile previous and recent findings to provide a compelling argument for the existence of a possible modifying role of loss-of-function in ALS [177]. They note that SOD1 activity is significantly reduced in ALS patients and that *SOD1*-null mice exhibit neuropathology similar to human ALS. Although a loss of SOD1 activity does not appear directly responsible for ALS phenotype, these data support the idea of a possible synergistic relationship between gain-of-function and loss-of-function in ALS disease progression. The interplay between gain- and loss-of function has also been described in a host of other neurodegenerative disorders, including Huntington’s disease and Parkinson’s disease [178,179]. As such, the implications to antisense therapy in neurology, and especially the antisense-mediated reduction of SOD1, are profound. The long-term effects of downregulating SOD1, therefore, should be an important focus of future clinical trials.

Isis Pharmaceuticals recently concluded a Phase 1 placebo-controlled, double-blind, dose-escalation, safety and tolerability clinical trial for their antisense drug ISIS-SOD1Rx (Table 1). The oligo employed in this study, ISIS 333611, was a 2'-MOE modified antisense oligo targeted to the first exon (19th–38th bps) of *SOD1* (regardless of mutation) and catalyzed RNase H1-mediated degradation [60,180]. The study involved patients from four US centers aged 18 years or older and carrying *SOD1* mutations. Participants were given 12-h intrathecal infusions of ISIS 333611 at varying concentrations, or placebo. No clinically significant adverse effects associated with oligo administration were reported. Following administration, AO was detected in the CSF of all AO-treated participants and increased with dosage concentration. SOD1 concentrations in the CSF did not change significantly, though achieving SOD1 reduction was never an aim of the study. 

In addition to the *SOD1* gene, several other genes have also been implicated in ALS pathogenesis, including the *TAR DNA binding protein* (*TARDBP*), *fused in sarcoma* (*FUS*), *angiogenin* (*ANG*), *ubiquilin 2* (*UBQLN2*), and *valosin-containing protein* (*VCP*) genes [181,182,183,184,185,186,187,188,189]. Most notably, it was recently discovered that a GGGGCC hexanucleotide repeat expansion in the first intron of the *C9orf72* gene is the most common genetic cause of ALS pathogenesis, more common than all other known ALS gene mutations combined, accounting for between 37%–50% of familial ALS cases among studied cohorts [190,191,192,193,194,195,196,197]. Although both loss-of-function and gain-of-function mechanisms have been postulated, the underlying etiology by which these *C9orf72* expanded repeats result in neurodegeneration is, as yet, unknown; however, evidence suggests a pathogenic threshold of hexanucleotide repeats may exist, though such a threshold has not yet been fully demarcated [191,192,195,198,199,200]. Because of the high prevalence of *C9orf72* mutations in cases of ALS, and because mutations in *C9orf72* have also been associated with other neurodegenerative disorders, such as Parkinson’s disease and frontotemporal dementia (FTD), *C9orf72* is a promising candidate for targeted antisense therapy [191,195,201,202,203]. Research groups are currently working with ISIS Pharmaceuticals to develop an antisense strategy for *C9orf72*-based ALS, working under the hypothesis that reducing mutant *C9orf72* transcripts using AOs will ameliorate toxic aggregations of expanded repeat mRNA, which present as nuclear foci in brain and spinal cord in affected patients [191,204]. Early investigations using AOs have yielded promising results, reducing the frequency of *C9orf72* expanded repeat aggregates and stabilizing gene expression *in vitro* [204,205].

## 12. Antisense Therapy for Huntington’s Disease

Huntington’s disease (HD) is an adult-onset, lethal, progressive neurodegenerative disease that follows an autosomal dominant pattern of inheritance. Clinical manifestations of HD include cognitive decay, such as the diminished ability to perform executive functions, motor deficits, such as chorea (involuntary, spastic movements), the inability to manage prehensile controls, and psychiatric disturbances, such as dysphoria, anxiety, irritability, mania and psychosis [206,207,208,209,210,211]. Neuropathological features of HD include widespread neuronal atrophy and the formation of nuclear/intranuclear inclusions in neural tissues of the brain [208,212,213,214,215,216,217]. Although the precise etiology of HD is still unknown, the disease is caused by a trinucleotide CAG-expansion in the first exon of the *Huntingtin (HTT)* gene, which results in a toxic gain-of-function of the resultant mutant huntingtin protein (mHTT) [218,219]. The inclusion bodies are composed of aggregates of misfolded mHTT and their density is highly correlated with repeat length [220,221,222]. Wild-type huntingtin (HTT) is ubiquitously expressed and is found at high concentration in the brain [223,224,225]. HTT is vital to proper embryonic development and neurogenesis, and also plays a role in protecting CNS cells from apoptosis, vesicular trafficking, axonal transport, and synaptic transmission [224,226,227,228,229,230,231,232,233,234,235]. Because the loss of HTT is associated with several deleterious consequences, the allele-specific silencing of mHTT is a promising therapeutic approach to treating HD [58,61,179,236], although some studies have shown significant beneficial effects from the co-suppression of both mutant and wild-type alleles [237,238,239].

The two foremost therapeutic approaches to allele-specific silencing of mHTT are the targeting of single nucleotide polymorphisms (SNP) and direct targeting of the expanded CAG region [240,241,242,243,244,245]. *In vivo* studies have demonstrated successful selective reduction of mHTT and a concomitant amelioration of HD neuropathology and behavioral/motor dysfunctions in mouse models [246,247,248]. Since AOs were first used to downregulate the expression of HTT, much attention has been given to developing antisense strategies aimed at selectively reducing mHTT levels [249]. A variety of AO chemistries, including PNA, LNA, 2'-MOE, and morpholino chemistries have been used *in vitro* and *in vivo* to selectively reduce levels of mHTT [58,239,243,245,250,251,252]. Notably, similar to DM1, 2'-MOE modified antisense oligo infusion into the cerebrospinal fluid of HD mouse models successfully reversed the disease progression with RNase H1-mediated degradation of huntingtin mRNA [239]. No clinical trials involving antisense oligos for the therapeutic treatment HD are currently being pursued; however, Prosensa Therapeutics is currently conducting preclinical tests of their antisense drug PRO289, designed to reduce levels of mHTT by targeting the expanded CAG tract (Table 1). So far, PRO289 has been successful in reducing mutant transcripts in HD patient-derived fibroblasts. 

## 13. Conclusions—What Does the Future Hold?

Lately, antisense therapies have moved one step closer to entrance into the clinical arena. The data from the Phase 2 DMD clinical trials are very promising. Isis Pharmaceuticals has recently started clinical trials of an antisense oligonucleotide therapy for SMA and ALS. Antisense drugs against FCMD, DM1, and Huntington’s disease are still in the preclinical stage of the development process but showed promising results in animal models. Some *in vitro* studies have demonstrated that dysferlinopathy is also a possible target for antisense therapy. Remaining challenges include limited delivery to the heart, potential off-target effects, lack of long term safety data, and limited applicability of each antisense oligo targeting each mutation (particularly in exon skipping therapy for DMD and dysferlinopathies). Unfortunately, the current regulatory process for drug development is not designed to handle these kinds of sequence-specific oligonucleotide therapies [253]. A re-evaluation of the current drug approval process, which takes into consideration the common characteristics of the same antisense chemistry and differences in the specific sequences, will help create a more efficient path for the development of antisense drugs and will benefit the progress of personalized medicine. 

With the recent clinical success of several antisense-based therapies, and establishment of proof-of-concept efficacy in several disease models, antisense oligos have established themselves as a promising and rapidly-developing therapeutic strategy covering a wide range of genetic disorders. With such dramatic improvements in antisense technology in a relatively short time frame, and with the current frenzied pace of antisense research, new and enhanced AO designs will likely be forthcoming and will facilitate their widespread application in the clinical realm.
